# Progress in Surgery

**Published:** 1899-09-16

**Authors:** 


### Progress in Surgery.

### OTOLOGY.

**Suppuration of the Middle Ear.**—Dr. Max Toplitz[1] discusses the diagnosis and treatment of these states. He describes three varieties of the disease, viz.: (1) Acute simple otitis media; (2) acute perforative otitis media, and (3) chronic suppurative otitis media. In acute simple otitis media the otoscope offers the picture of inflammation of the membrana tympani, which is frequently bulged by the pressure, particularly in the postero-superior quadrant. There is pain in the ear, fever, headache, often toothache. In infants, violent cerebral symptoms have been observed, and in suspected cerebral disease in children the ears should be examined. Infants with it are restless, cry incessantly, and grasp the head, are feverish, and become emaciated. The perforative otitis offers the same picture with a perforation of the drum, and with secretion seen at the bottom of the meatus, which is serous, sanguinolent, purulent, or muco-purulent. The symptoms are the same as in the last form, but more severe, and may entirely disappear after the establishment of the perforation. In certain states chronic otitis media follows the above acute inflammations, *e.g.*, in scarlet fever, diphtheria, typhoid fever, and in scrofulous, syphilitic, and cachectic individuals, and in chronic affections of the nose. In the treatment, above all, proper drainage must be secured. The perforation, whether natural or artificial, must be enlarged if too small. Permanent openings are produced by the galvano-cautery. Astringent injections, like sulphate of zinc, are to be avoided in acute cases, and boric or carbolic acid (1-2 in 100), or sublimate (1 in 6,000 to 8,000) used. Afterwards dry the meatus and close with sterilised gauze. Later a thin layer of iodoform or aristol may be applied by Urbanschitsch's powder blower. Irrigations through the Eustachian tube are not recommended, for the entrance to the mastoid cavity lies in the line of that tube, and bacteria may be carried in from the naso-pharynx. Grenning's method of suction is recommended if the secretions in the middle ear are tenacious. The alcohol treatment of Politzer is very valuable. Begin with instillation of a saturated solution of boric acid in 25 per cent. alcohol, and gradually increase the strength of the alcohol even to absolute alcohol if well borne. Peroxide of hydrogen is useful. It forms bubbles of oxygen when injected, which tend to cleanse recesses. If the above treatment fails, operations are necessary. Small granulations are to be cauterised with chromic acid fused on a probe or holder. Large polypi removed with the snare, sharp spoon, or ring knife. Nasal obstructions must be cured. Ossiculectomy is indicated, if they are carious. Tiny fistulæ form in the drum in such cases. The operation is not easy, and has often to be done by tactile sensations only. If these methods fail the mastoid process must be operated upon.

**Mastoid Disease.**—Cazzolino in 1894 called attention to the speedy reappearance of pus in the tympanum as the "unique symptom" of endomastoid suppuration, and in 1895-6 pointed out that the pus followed a definite course in the tympanum. It always flows in a single line over the inner wall of the cavity from the postero-superior to the postero-inferior segment, passing in front of the fenestra ovalis and rotunda when the head is vertical. Barrago-Ciarella[2] now gives details of six cases in which "Cazzolino's symptom" was the only, but unfailing, sign of pus in the mastoid, and claims that the sign is pathognomonic. Ménière[3] relates a case of mastoiditis which followed an otitis, which had been rapidly cured by dilating the perforation in the drum, and other treatment. Pain returned, and was located three or four centimetres behind the point of the mastoid. There was some swelling and crackling in the ear when pressure was made on the mastoid. A carious focus was found low down in the apophysis, and from this a fistulous track was traced up to the antrum. The condition was cured with intact membrane and normal hearing. Dr. Gorham Bacon[4] reports a case of mastoid disease affecting both sides with symptoms suggesting intracranial complications. The patient, a boy of four, had severe pain in the left ear, and a temperature of 103 deg. to 104 deg. Fahr. Both drumheads were bulging and red, and the left had a perforation. This was on the fifth day of pain, and Dr. Bacon incised both membranes. The temperature continued and the boy became dazed, so that three days later the left antrum was opened. It contained softened bone and granulations. A few days later the right antrum was operated upon. The outer walls of the sigmoid sinuses were exposed. They were thickened and red. After the last operation there was rapid recovery. Pneumococci, with some streptococci, were found in the discharges. Dr. Bacon advises against exploring the sinuses with a needle, as it is of little value and may infect the sinus. A free incision is the only reliable method. There might be a thrombus, even if the needle withdrew fluid blood. Morton[5] also reports a case of bilateral mastoiditis, and refers to the smallness of the number of such cases. The patient, a woman of 54, had pain in and discharge from both ears. There was redness over and tenderness of both mastoid processes. Both antra were subsequently opened and pus removed. Rapid recovery without deafness ensued. Betham Robinson[2] records a case of squamous-celled epithelioma following chronic otitis and mastoiditis. A woman of 46 had had otorrhœa for 26 years. It had become more profuse four years before, and deafness, with noises in the head and shooting pains, came on then. The mastoid was exposed, and nearly the whole of it found to be softened and excavated. A month later the swelling and infiltration over the mastoid were much more marked, the edges of the wound being everted, and the meatus blocked with granulations. A portion from over the mastoid was shown by the microscope to be a squamous-celled epithelioma. There was fulness in the parotid region. Probably the tumour arose from the deep part of the external meatus, and as a result of prolonged irritation.

**Extra Dural Abscess.**—Dr. Milligan[6] reports two cases of this condition, in which, after operation, recovery ensued. The most usual site of these abscesses, which may be quite small or contain several ounces, are over the tegmen tympani, or in the neighbourhood of the sigmoid groove. In the latter case thrombosis of the sinus is very prone to occur. The symptoms may be slight or severe, and resembling abscess of the brain or meningitis. In Milligan's first case, that of a man of 48, there had been four weeks' pain, with deafness and noises in the ear. The membrana tympani was intensely congested. The mastoid was tender. The drum was incised, and copious discharges followed in the next few days, but the pain and temperature remained high. The antrum and posterior fossa of the skull were accordingly explored. From the latter about an ounce of pus escaped. There was a small quantity only in the antrum. Recovery with normal hearing power was the result. The second case was a man of 20, who had had discharge from both ears from childhood. For some months he had had pain over the left side of the head. When Milligan first saw him there was an œdematous and boggy condition of the tissues behind the mastoid. This was incised, and a larger quantity of fœtid pus escaped. A fistula the size of a sixpence passed through the cranial wall, at the posterior border of the mastoid, into a large extra-dural abscess. The antrum was practically normal. A rapid recovery was made. Professor Cazzolino records three cases of extra-dural abscess. In one case, a boy, the symptoms of otitic inflammation were from the first associated with those of endo-cranial mischief, and there were no cutaneous or external symptoms of mastoiditis. There was pain from the middle of the head to the frontal region. When the external mastoidal wall had been broken down by operation a natural dehiscence of the internal wall of the cavity corresponding to the sulcus for the sigmoid sinus was exposed, and pus flowed from it with pulsations. The wall of the sigmoid sulcus was therefore broken down, and all the pus evacuated which lay on the cerebellar dura mater. Recovery ensued. This case is cited as showing again that otitic infections, when they invade the cranial cavity, always do so through openings, either pathological, as from osteitic processes of the surfaces of the petrous bone, or else by natural dehiscences from arrest of ossification. These conclusions have been confirmed by experimental pathology, as no one has as yet been able to produce the intracranial complications of ear disease by means of infection of the middle ear in animals. In another case, succeeding otitis due to sea bathing, there was mastoiditis followed later by retro-auricular swelling, with vertigo and fever. A subcutaneous abscess was found between the occiput and mastoid, and a necrotic portion of the occipital bone was found on opening this. The dura was laid bare by curetting the necrosed bone. The pus did not issue in jets, being below the level of the sinus. This is an example of cranial extension in an acute case of otitis, which is only exceptionally observed. It is remarked that the symptoms of extra-dural abscesses are rarely typical, and very often run their course without any marked characters which distinguish them from pure endo-mastoiditis with empyema. They may, again, simulate lepto-meningitis, from the pus being under great pressure and having no outlet. The pulsatile flow of pus from the internal wall of the mastoid cavity, owing to the impulse from the sinus and its quick reappearance after wiping away, are important signs of these collections. Their treatment obviates the danger of the graver intracranial complications occurring.

[1] Med. Rec., Dec., 1898. [2] Jour. of Laryngol., Rhinol., and Otology, Mar., 1899. [3] Jour. of Laryngol., Rhinol., and Otology, April, 1899. [4] Jour. of Laryngol., Rhinol., and Otology, June, 1899. [5] The Laryngoscope, Feb., 1899. [6] Jour. of Laryngol., Rhinol., and Otology, Jan., 1899. [7] Jour. of Laryngol., Rhinol., and Otology, May, 1899.

### ORTHOPÆDIC SURGERY.

(*Continued from page* 406.)

**Congenital Talipes Equino-Varus.**—Dr. A. F. Jonas[1] remarks that the management of talipes equino-varus tests the skill and practice of the surgeon as perhaps no other surgical disease. A modification is here described which may prove useful—finding a rapid route for the successful and immediate correction of inveterate varus deformities. It seems that if an attached flap with a broad pedicle can be produced that under all circumstances will contain a sufficient blood supply to insure its life, and at the same time cover the greater part of the wound, more particularly the deeper part of the wound produced by the severed fascia, tendons, muscles, and ligaments, we shall have made an advance in the direction of preventing recurrence caused by contraction of the soft parts. It is now proposed to make a triangular flap similar to that used in relieving Dupuytren's contraction of the fingers, a V-shaped incision being made as advocated by Busch, and by others. An incision is made, beginning slightly below the margin of the plantar fascia on the inner side of the foot, at a point on a line directly below and anterior to the internal malleolus, extending forward and upward to a point on the first metatarsal bone, and nearly to the metatarso-phalangeal articulation. A second incision is made, beginning at a point over the astragalo-scaphoid articulation, extending forward and slightly downward, joining the first incision near the metatarso-phalangeal joint, forming a V. The incisions are made deep, so as to include the subcutaneous tissue and fat. This flap is dissected backward to the points first indicated. The shortened soft structures are now all exposed. The inner fasciculus of the plantar fascia is severed first. The diagonal division of the plantar fascia is done, so that after correction there shall not be left a defect between the divided ends, but that the points of the incised fascia still come in contact, thereby lessening the tendency to contraction of this structure. The remaining structures are now divided successively, as directed by Phelps, until the astragalo-scaphoid capsule is reached. Instead of dividing this another incision is made on the outer side of the foot over the head of the astragalus, pushing aside the tendons and soft structures, and exposing the neck of that bone and then cutting through the neck with a chisel. The forward part of the foot can now be pushed outward without separating the astragalo-scaphoid articulation which nearly always occurs in the typical Phelps's operation. Occasionally, however, in old inveterate cases it becomes necessary to remove the head of the astragalus. The foot is over corrected, the varus being turned into a valgus. The equinus position can now be relieved by subcutaneous division of the tendo-Achillis. If there is bleeding from the wound on the inner side of the foot it can be controlled by catgut ligature. The triangular flap is then turned back, it covers the wound except at its anterior point. A perforated silk protective covers this wound. The outer wound, over the head of the astragalus, is closed with catgut. An antiseptic dressing is applied, and over this a retentive dressing of plaster-of-Paris, beginning at the toes, extending above the knee, including one-third of the length of the thigh. It is left undisturbed for five or six weeks, and on removal of the dressing the wounds will have completely healed. This method has been employed 25 times, only in old, inveterate, and relapsing cases, and has been found more satisfactory than other methods previously employed. But observation should be insisted on for several years, because a partial recurrence will take place unless suitable retentive apparatus is employed. The advantages of the triangular flap are: The flap, on account of its broad attachment, is not likely to slough; it is thick, and more completely fills up the large, gaping wound than any other flap; the defect left underneath the flap fills with a blood-clot facilitating connective tissue formation by gradual displacement of the blood elements. The healing process is identical with that of the blood-clot as first produced in bone cavities by Schede, and which he called organisation of the thrombus. There is no broad and deep granulating surface causing abrupt and shelving sides.

The advantages of dividing the plantar fascia diagonally from before backward are: No dead space is left between the ends of the severed fascia; the points of the divided fascia still touch, and can be sutured if deemed advisable.

[11] Annals of Surgery, June, 1899.

### GENERAL SURGERY.

**Asepsis in Surgery.**—It is now generally granted that the chief danger of infection in aseptic operations, as J. Lynn Thomas states, is from the hands of the operator, his assistants, and nurses, because while instruments, dressings, &c., can be rendered sterile with absolute certainty, it is otherwise with the hands. Therefore some surgeons do not touch wounds with their hands, but endeavour to perform every possible manipulation by means of instruments alone. Lynn Thomas[1] has added his experience to that of Mikulicz, Manteuffel, McBurney, A. Wölffler, and others in using gloves in aseptic operations. For many months he has tried gloves made of silk, taffeta, thread, and cotton, but likes best the buttonless thread gloves, because they are closely woven, fit well, stand any amount of sterilisation, and cost but a few pence per pair. The subdued sense of touch does not in any way interfere with the due performance of the manipulations in operating. Since he has taken to gloves he feels greater confidence in prophesying with regard to the healing of wounds than he did before adopting them. The most striking result from the wearing of gloves in his aseptic cases has been the total disappearance of suture abscesses, and even of the suture blush in every case except two. As to the use of the nail-brush in surgical asepsis, Geo. T. Beatson[2] asks whether it is not carrying the mechanical removal of septic matters too far, and whether harm may not sometimes be done both to patient, the surgeon, and his assistants by this interference with that protection against infection which the normal and intact skin affords. He is of opinion that many of the troublesome suppurations of the fingers that happen to surgeons, students, and nurses are due to scrubbing the hands with nail-brushes after exposure to pyogenic organisms, thereby forcing these latter into the tissues. And this can be done even with aseptic nail-brushes. In the same way he believes that the violent measures taken by many for the purification of the patient's skin may be the exciting cause of the often inexplicable stitch abscesses that from time to time arise. A Russian surgeon, Dr. Cirikow,[3] believes that the hands will beyond all doubt be disinfected if cleansed by mechanical means during three minutes, followed by brushing with a new brush in 95 per cent. alcohol during three more. They will then even remain aseptic after steeping in hot water during twelve minutes, or after four or five minutes of perspiration. Alcohol should never be used under 80 per cent., and even at that degree the results are only semi-satisfactory. Wood spirit at 92 per cent. is very cheap, and gives excellent results. The thread gloves used by Mikulicz, he thinks, have little value. A consideration generally passed over in silence in works treating of aseptics and antiseptics is important, viz., the maintenance in an aseptic condition of all solids and liquids which may come into contact with bleeding surfaces, from the moment the destruction of germs has been accomplished until the dressing of the wound is perfected. R. B. Duncan[4] draws attention to this in a recent paper on the sterilisation of the different articles used as dressings. It is now a universally admitted principle that all dressings used for wounds must be perfectly pure of all pathogenic germs. The micro-organisms of the air have been recognised as inoffensive, no doubt, on account of their number being kept in check by the phagocytes, but infection by contact is formidable, and unexpected accidents, too often for the need of complete asepsis, confirm every day the absolute necessity of perfect sterilisation. Heat is the sole means of removing from dressings all cause of contagion, and employed to a sufficient degree destroys all living beings, vegetable or animal, and their germs. It destroys equally soluble ferment diastase enzymes, toxines which we know resist antiseptics. As M. Vinay remarks, another consideration should lead us to prefer heat to the use of antiseptics; this is the uncertainty which still exists of the resistance of contagious diseases, like small-pox, scarlatina, measles, and whooping cough. The contagious nature of these exanthema is doubted by no one, but their parasite remains unknown. In concluding an address given before the Manchester Clinical Society, on "Aseptic *versus* Antiseptic Surgery," T. R. Jessop[5] tersely sums up the subject as follows: Antiseptic surgery rightly assumes that the patient's skin, the surgeons' and nurses' hands, the instruments and sponges, the ligatures, sutures, and dressings are all sources of septic infection, and need for their purification a more or less prolonged exposure to some germ-destroying agent. It errs, perhaps, in its estimate of the danger resulting from a short exposure of the fresh wound to the atmosphere, and in its non-recognition of Nature's power to deal with septic organisms when in such minute quantity as ordinarily obtains during such exposure, and in inculcating the need for their destruction by the lavish use of powerful chemical agents, themselves not wholly innocuous. Aseptic surgery, whilst recognising the necessity for destroying the vitality of such putrefactive organisms as have access in appreciable quantity to the wound, leaves the natural forces to work repair, unhampered by the presence of chemical substances, and is satisfied to preclude the introduction of any fresh deleterious matter by means of a filtering material, so applied as completely to cover the wound and its neighbourhood.

[1] Brit Med Jour. Jan. 21, 1899, p. 140. [2] Ibid., Feb. 4, 1899, p. 316. [3] Lancet, Feb. 25, 1899, p. 531. [4] Intercolonial Med. Jour., Feb. 20, 1899 p. 120. [5] Med Chron., June, 1899, p. 189.

